# Sodium selenate biofortification, through seed priming, on dill microgreens grown in two different cultivation systems

**DOI:** 10.3389/fpls.2024.1474420

**Published:** 2024-11-27

**Authors:** Hossein Sheikhi, Silvana Nicola, Mojtaba Delshad, Roberta Bulgari

**Affiliations:** ^1^ Horticultural Sciences Department, College of Agriculture and Natural Resources, University of Tehran, Karaj, Iran; ^2^ Department of Agricultural, Forest and Food Sciences (DISAFA), University of Turin, Grugliasco, Italy

**Keywords:** microgreens, *Anethum graveolens* L., soilless cultivation systems, light emitting diodes, vegetables quality, biofortification, mineral elements

## Abstract

Human health is significantly influenced by the quality of vegetables included in the diet. Soilless cultivation methods have the potential to enhance and standardize the levels of secondary metabolites or specific bioactive compounds in plants, even when utilizing LED lighting. In recent years, tailored foods, enriched with important microelements, are growing in popularity. The present research was conducted to explore the quantitative and qualitative aspects of dill (*Anethum graveolens* L.), grown either indoor or in a greenhouse and harvested during the microgreen stage. Seeds of dill were primed with 1.5 and 3 mg L^−1^ selenium (Se). Untreated dry and hydro-primed seeds were used as the control and positive control groups, respectively. Results demonstrated a higher yield in indoor farm environment (1255.6 g FW m^−2^) compared to greenhouse (655.1 g FW m^−2^), with a general positive effect on the morphological traits studied, with no significant influence from priming and Se. The mean value of phenolic index of microgreens grown in the greenhouse was 13.66% greater than that grown in indoor condition. It was also observed that seeds priming with Se can effectively raise the Se content in dill microgreens, in both tested conditions. Overall, our results suggest that the 3 mg L^−1^ Se seems to be the most promising concentration to obtain Se-enriched microgreens.

## 1 Introduction

Among innovative vegetable products, microgreens have played an important role in recent years (Lone and Pandey, 2024; Bhaswant et al., 2023; Mezeyová et al., 2022). They are very suitable to be grown under controlled conditions, on a growing medium, in the presence of natural or artificial light, and require little or no fertilizers (Gao et al., 2021). They are generally ready to eat 7-21 days after seed germination, depending on plant species and growing conditions (Bulgari et al., 2021; Lenzi et al., 2019). The higher concentration of bioactive molecules and mineral nutrients contained in microgreens, compared to mature plants, has contributed to their growing appreciation by modern consumers (Lone and Pandey, 2024; Brazaitytė et al., 2021; Kyriacou et al., 2021), in addition to the interest of producers as they represent a very profitable product. Microgreens are used to give distinctive flavors to a variety of dishes, to embellish salads, sandwiches, and soups with their diverse colors and shapes (Bhaswant et al., 2023; Teng et al., 2023; Bulgari et al., 2021), as well as flavors and textures. The relative simplicity of their cultivation, the shortness of their growing cycle, and the minimal space requirement are encouraging the spread of this type of cultivation in indoor systems (vertical farming) within urban and peri-urban areas, as well as on spaceships (Tavan et al., 2024; Amitrano et al., 2023) to provide fresh vegetables with nutraceutical properties to astronauts during long space missions. Techniques such as biofortification may further increase the nutraceutical potential of microgreens (Newman et al., 2021; Pannico et al., 2020). The use of micronutrients such as selenium (Se), iodine, zinc, iron, etc., to biofortify young vegetables is becoming more popular (Viltres-Portales et al., 2024; Poudel et al., 2023; Ciriello et al., 2023; Guardiola-Márquez et al., 2023; Mezeyová et al., 2022; Puccinelli et al., 2021; Newman et al., 2021) and plays an important role in improving the daily intake of critical macro and micronutrients in adults and children. Since Se is essential for human health as it plays a significant role in antioxidant defense, biofortification with this element represents a good agronomic strategy for obtaining microgreens with a higher nutritional value.

For the biofortification of plants, foliar spraying, soil application, and direct delivery of Se in nutrient solution are often employed methods (Izydorczyk et al., 2021), but since microgreens have a short growth period and do not have a high leaf area, these techniques may not be effective. It has been found that nutri-priming is associated with improved germination and seedling growth (Khaliq et al., 2015). In this technique, instead of pure water, the seeds are soaked in solutions containing limited nutrients (Lutts et al., 2016). Since nutri-priming is applied to seeds, it eliminates the possibility of adding harmful chemicals to the environment (Bhatia and Gupta, 2022). Additionally, it is an uncomplicated, economical, and innovative strategy that can serve as a sustainable alternative to fertilizers and agrochemicals. The positive effects of Se seed priming on germination, seedling development, and biochemical attributes have been documented in some crops (Shahverdi et al., 2017; Khaliq et al., 2015).

With regard to cultivation systems, as reported before, microgreens have been shown to perfectly fit in indoor growing environments and are therefore suitable for small and large-scale production. Researchers have conducted many studies on microgreens in controlled systems, while comparing these different typologies of systems in their production has received less attention (Brazaitytė et al., 2021; Bulgari et al., 2021; Gao et al., 2021; Puccinelli et al., 2021). Specifically, biofortification of dill (*Anethum graveolens* L.) with Se through priming under these advanced cultivation systems has not been studied. Therefore, the present research aims to investigate the possibility of Se biofortification of dill microgreens through priming under greenhouse and indoor farm systems.

## 2 Materials and methods

### 2.1 Plant growth, treatments tested, and harvest

The present experiments were carried out at the Department of Agricultural, Forest and Food Sciences (DISAFA), University of Turin, Grugliasco, Italy (45°03′59.73″N, 7°35′24.72″E). Two separate trials on biofortification of dill microgreens with selenium (Na_2_SeO_4_) through priming were conducted, under greenhouse (E1) and indoor farm (E2) conditions. The cultivation took place as follows, and as reported in a preliminary test described by Bulgari et al., 2022. Dill seeds from Cooperativa Ortofrutticola Srl, Albenga, Italy, underwent disinfection process using 5% of sodium hypochlorite for 5 min, followed by thorough washing with distilled water, repeated 10 times. The seeds were primed at room temperature (RT) for 8 h in selenium solution at 1.5 and 3 mg L^-1^ concentrations. For hydro-priming, the seeds were immersed in distilled water for an equal duration. Unprimed dry seeds were considered as control. Following each treatment, the seeds underwent three washes with distilled water and were subsequently dried in an oven at 44°C for 15 h, until they reached the initial moisture level. Afterward, 1 g of dill seeds were uniformly sown in aluminum boxes (130x70x40 mm) filled with hemp-pad substrate, which was chosen because it is a typical product of the area where the cultivation took place and therefore also easy to find. The disinfection of the hemp was carried out using a 5% sodium hypochlorite solution and then rinsing with distilled water. The seeds were sown and immediately transferred to the experimental glasshouse, for E1, under monitored conditions (23°C, 35% RH, 11.3 MJ m^-2^, and 12 h photoperiod, as average values during the experimental period). They were irrigated with distilled water until complete germination. Ten days after sowing, seeds were irrigated using a 40/60 N-NO_3_^−^/NNH_4_^+^ nutrient solution, composed of (all in mmol L^−1^): 6 N, 2 P, 6 K, 2 Mg and 2.5 Ca. During the experiment, a total of 5.2 L of nutrient solution was used. Microgreens were harvested 25 days after sowing. They were collected at the base, approximately 5 mm from the growing pad.

In the E2, the seeds preparation and treatments were the same of the E1, except that the boxes were transferred to a small indoor farm (Microtype:<3 m^3^ volume). Temperature and relative humidity of the indoor farm were 24°C and 55%, respectively. The indoor farm was equipped with LED lights. The LED spectrum consisted of 24% blue, 15% green, 56% red and 11% far-red, with a PPFD of 225 µmol m^−2^ s^−1^ and a light/dark period of 14/10 h. The amount of nutrient solution used in this experiment was the same as the E1 (5.2 L). In the trial, microgreens were also harvested after 25 days, in the same way as previously reported.

For each experiment, 4 treatments were applied with 3 replications, and 2 aluminum boxes for replication were used. From each replication, 1 aluminum box was selected to measure the morphological traits, immediately at harvest. In order to perform biochemical assays, the remaining plant material was stored at -80°C, until the time of determination.

### 2.2 Plant growth estimation (height, FW, yield, DW, DM%)

The growth parameters of dill microgreens, such as the plant height and the fresh weight (FW), were measured at harvest, which occurred 25 days after sowing. The yield of microgreens was indicated in g m^-2^. After these measurements, the samples were transferred for 3 days into an oven with a constant temperature of 50°C, to calculate the dry weight (DW). After that, the dry matter percentage (DM%) was calculated.

### 2.3 Mineral content determination

For the mineral elements determination, dried microgreens were digested in wet conditions [HNO_3_ 67% and H_2_O_2_ concentrated (30%)]. In detail, 0.25 g of sample were digested with a mixture of 8 mL of nitric acid and 2 mL of hydrogen peroxide, in microwave (method ISO 11466 - Milestone Ethos D, Sorisole (BG), Italy). The digested sample was then filtered with cellulose filters (Whatman Grade 41) and made up to volume to 50 mL with deionized water. The mineral elements (K, Mg, and Se) were then read, with appropriate dilutions, by ICP-MS (Perkin-Elmer NexION 350D, Waltham, MA, USA). The accuracy was checked using Standard Reference Materials for plant (NIST SRM 1572, tomato leaves, National Institute of Standards and Technology, Gaithersburg, MD, USA) and all recoveries of analyzed mineral elements were between 90% and 110%. All reagents used in the determination were of an ultrapure or analytical grade.

### 2.4 Color values

Microgreens canopy colors (L*, a*, b*, Chroma, and hue angle) were measured, *in vivo*, at three different points on each aluminum box, using a Spectrophotometer CM-2600 (Konica Minolta Sensing Inc., Osaka, Japan). L* corresponds to lightness or brightness, while redness, greenness, and yellowness are expressed as +a*, -a*, and b*, respectively. Hue angle ( $\overset{\text{o}}{\text{h}}$ ) and Chroma (C*) were calculated using the following equations, according to McGuire (1992):


$${\text{h}}^{{}^{\circ}}=\tan^{-1}\frac{{\text{b}}^{*}}{{\text{a}}^{*}}$$

$$C^{*}=\left[{\left(a^{*}+b^{*}\right)}^{1/2}\right]$$

### 2.5 Pigments determination

Regarding chlorophylls and carotenoids determination, 50 mg of frozen microgreen dill tissue were extracted with 100% methanol (v/v) and then kept at 4°C for 24 hours in a dark room. Absorbance for chlorophyll *a*, *b*, and carotenoids was read at wavelengths of 665.2, 652.4, and 470 nm, respectively, using a UV–Vis spectrophotometer (Cary 60 UV-Vis, Agilent Technologies, Santa Clara, CA, USA). Based on the FW of the tissue, Lichtenthaler’s formula (Lichtenthaler, 1987) were used to calculate the pigments concentration.

### 2.6 Phenolic index, total sugars, °Brix, and nitrate concentration

Phenolic index was determined from 30 mg FW of dill microgreens. Samples for each treatment were transferred to a tube containing 3 mL of methanol acidified with hydrochloric acid (1% v/v) and were kept in a dark room for 24 h at 4°C. Absorbance readings were determined with a spectrophotometer at 320 nm (Ke and Saltveit, 1989). Phenolic index was expressed as ABS320 nm g^−1^ FW. For sugars, an aliquot of 1 g of fresh tissue was homogenized in 3 mL of distilled water and centrifuged at 4000 *x* g for 15 min at RT (Benchtop centrifuge - Hettich, model ROTANTA 460 R, Tuttlingen, Germany). The total sugar levels were assayed according to the anthrone assay (Yemm and Willis, 1954). Absorbance was read at 620 nm and the concentration was calculated referring to a glucose calibration curve. The soluble solids content was determined using a portable digital refractometer (Hanna HI 96801, Hanna Instruments, Smithfield, England), using the same aqueous extract prepared for the total sugars assay and previously described. Nitrates content was determined with the salicylsulphuric acid method (Cataldo et al., 1975). The aqueous extract used for the analysis was the same as the preparation for the determination of sugars. Twenty µL of sample were added to 80 μL of 5% salicylic acid in sulphuric acid and to 3 mL of NaOH 1.5 N. Samples were cooled at RT for 15 min and the spectrophotometer readings were performed at 410 nm. Nitrate concentration was calculated referring to a KNO_3_ standard calibration curve (0, 1, 2.5, 5, 7.5, 10 mM KNO_3_).

### 2.7 Statistical analysis

Statistical analysis was performed using SAS software version 9.4 (SAS Institute, Cary, NC, USA). The two experiments were carried out independently each as a completely randomized design with 4 treatments and 3 replications. The comparison of means was evaluated with the least significant difference test (LSD, p=0.05). The normality of the data was checked by the Kolmogorov-Smirnov test. The t-test was used to determine the significant difference between the means of E1 and E2. Using GraphPad Prism 9.5.1 (GraphPad prism, Prism for Windows, version 9.5.1), a heatmap was created for yield, phenolic index, nitrate, and Brix°, as well as mineral elements. Principal Component Aanalysis (PCA) and correlations among the traits were performed using the R Studio 2022 software version 4.2.1 (R Core Team, Development Core Team, 2020).

## 3 Results

### 3.1 Estimation of plant growth

**Figure 1:**
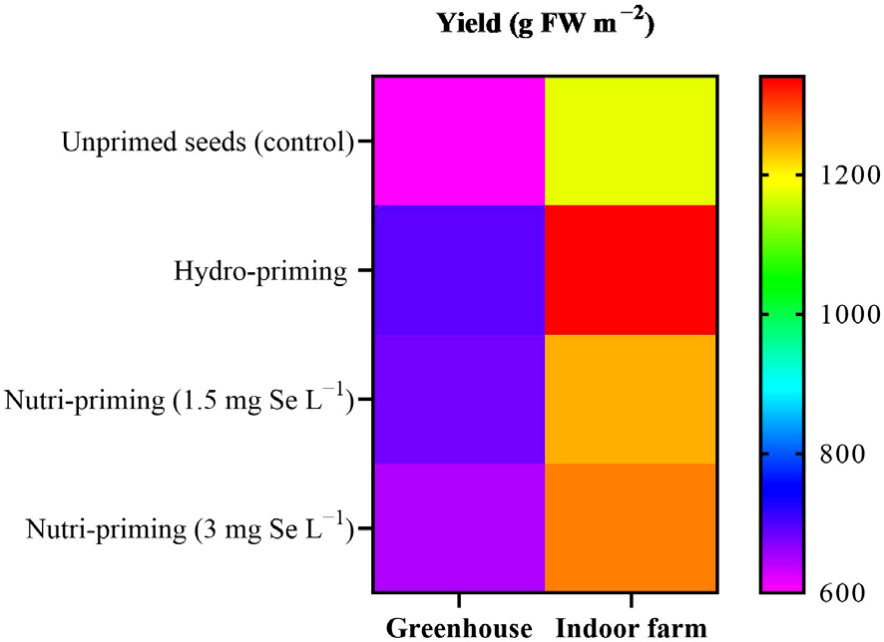
Visual representation of yield trends among treatments as well as between greenhouse and indoor farm conditions.

Based on the results of the ANOVA analysis, it was found that the yield of dill was not significantly affected by seed priming with Se in greenhouse and indoor farm systems. It was observed that seed priming, especially hydro-priming, enhanced the mean value of yield of dill microgreens compared with seeds that had not been primed (**Table 1**). Moreover, seed priming improved the biomass of dill microgreens in both experiments, although this difference was not statistically relevant (**Table 1**, **Figure 1**), with a more pronounced effect in indoor conditions, where yields were higher. The results revealed that Se seed nutri-priming positively affected the trend of the DM%, so its effect was greater than hydro-priming in terms of value. Under indoor farm conditions, nutri-priming with 3 mg Se L^−1^ was more effective than 1.5 mg Se L^−1^, increasing the DW and FW but, under greenhouse conditions, nutri-priming with 1.5 mg Se L^−1^ was more effective. In both studied growing systems, seed priming with 3 mg Se L^−1^ had a greater effect on DM% than 1.5 mg Se L^−1^, although not significantly. There was also no significant difference in the height of dill microgreens in both growing systems in terms of its response to seed priming (**Table 1**).

**Table 1:** Morphological traits of dill microgreens treated with different levels of Se-priming in both greenhouse and indoor farm system.

Cultivation system	Treatments	Yield (g FW m^−2^)	Dry weight (g)	Fresh weight (g)	Dry matter (%)	Height (cm)
Greenhouse	Unprimed seeds (control)	599.82 ± 28.7	0.79 ± 0.02	5.45 ± 0.26	14.22 ± 0.39	3.27 ± 0.14
	Hydro-priming	692.31 ± 31.5	0.92 ± 0.04	6.30 ± 0.28	14.96 ± 0.40	3.46 ± 0.03
	Nutri-priming (1.5 mg Se L^−1^)	679.85 ± 16.3	0.92 ± 0.03	6.18 ± 0.14	14.63 ± 0.68	3.75 ± 0.38
	Nutri-priming (3 mg Se L^−1^)	648.35 ± 40.9	0.89 ± 0.02	5.90 ± 0.37	15.52 ± 0.93	3.44 ± 0.29
Significance		ns	ns	ns	ns	ns
Indoor farm	Unprimed seeds(control)	1173.2 ± 37.4	1.48 ± 0.04	10.67 ± 0.34	13.92 ± 0.06	6.06 ± 0.13
	Hydro-priming	1340.8 ± 45.1	1.49 ± 0.06	12.20 ± 0.41	12.21 ± 0.35	6.37 ± 0.03
	Nutri-priming (1.5 mg Se L^−1^)	1241.4 ± 16.3	1.54 ± 0.003	11.28 ± 0.14	13.67 ± 0.20	6.22 ± 0.19
	Nutri-priming (3 mg Se L^−1^)	1266.8 ± 53.3	1.57 ± 0.02	11.52 ± 0.48	13.73 ± 0.74	6.22 ± 0.09
Significance		ns	ns	ns	ns	ns

ns, indicates non-significant. All data are expressed as mean ± standard error (SE), n=3. The least significant difference test (LSD) was used to compare the means of treatments for each trait (p=0.05).

The t-test results showed a significant difference among these measured parameters under different growth systems (**Table 2**). The plants grown in indoor conditions performed better than in greenhouse, concerning yield, FW, DW, and height. Based on the results of the present study, an increase of 91.66% in the yield of microgreens was found in indoor farm, with an average yield of 1255.6 g FW m^−2^, compared to greenhouse (average yield of 655.1 g FW m^−2^) (**Table 2**). Regarding FW and DW, the indoor farm produced microgreens with 91.61% and 72.72% increase in FW and DW, respectively, compared to those grown in the greenhouse, while their DM% decreased by 9.77% (**Table 2**). The results of the t-test also showed that the height of the microgreens grown in indoor farm, with an average of 6.22 cm, was nearly twice the height of those grown in greenhouse, with an average of 3.48 cm (**Table 2**).

**Table 2:** Results of independent t-test for comparison between greenhouse and indoor farm system for all measured traits.

Variables	Pr > |t|	Greenhouse		Indoor farm	
		Means	SE†	Means	SE
Yield (g FW m^2^)	<.0001	655.1	16.9	1255.6	24.9
Dry weight (g)	<.0001	0.88	0.02	1.52	0.02
Fresh weight (g)	<.0001	5.96	0.15	11.42	0.22
Dry matter (%)	0.0021	14.83	0.31	13.38	0.27
Height (cm)	<.0001	3.48	0.41	6.22	0.22
L*	0.0090	26.34	4.6	30.87	2.9
a*	0.0022	-5.62	2.3	-8.27	1.2
b*	0.0003	17.34	0.81	21.69	0.62
Chroma	0.0004	18.31	3.3	23.24	2.4
Hue	0.0207	72.72	0.02	69.18	0.00
Chlorophyll *a* (μg mg^−1^ FW)	0.0003	0.63	0.02	0.75	0.01
Chlorophyll *b* (μg mg^−1^ FW)	0.3368	0.23	0.00	0.22	0.01
Total Chlorophylls (μg mg^−1^ FW)	0.1619	0.87	0.02	0.80	0.03
Carotenoids (μg mg^−1^ FW)	0.2125	0.16	0.00	0.15	0.00
Chlorophyll *a*/*b* ratio	0.2087	2.66	0.06	2.56	0.05
Chlorophylls/Carotenoids ratio	0.9848	0.18	0.00	0.18	0.00
Phenolic index (ABS_320_nm g^−1^)	0.0448	29.20	1.1	25.69	1.1
Total sugars (mg kg^−1^ FW)	0.3497	4912.9	249.9	5335.1	364.4
Nitrate (μg g^−1^ FW)	0.0005	328.2	45.6	108.9	12.9
°Brix	0.0093	1.11	0.01	1.00	0.03
K (mg g^−1^ DW)	<.0001	7.50	0.24	45.48	2.3
Mg (mg g^−1^ DW)	0.0020	3.85	0.29	2.66	0.04
Se (µg g^−1^ DW)	0.1016	0.581	0.08	0.39	0.06

†SE represents the standard error.

### 3.2 Mineral concentration

**Figure 2:**
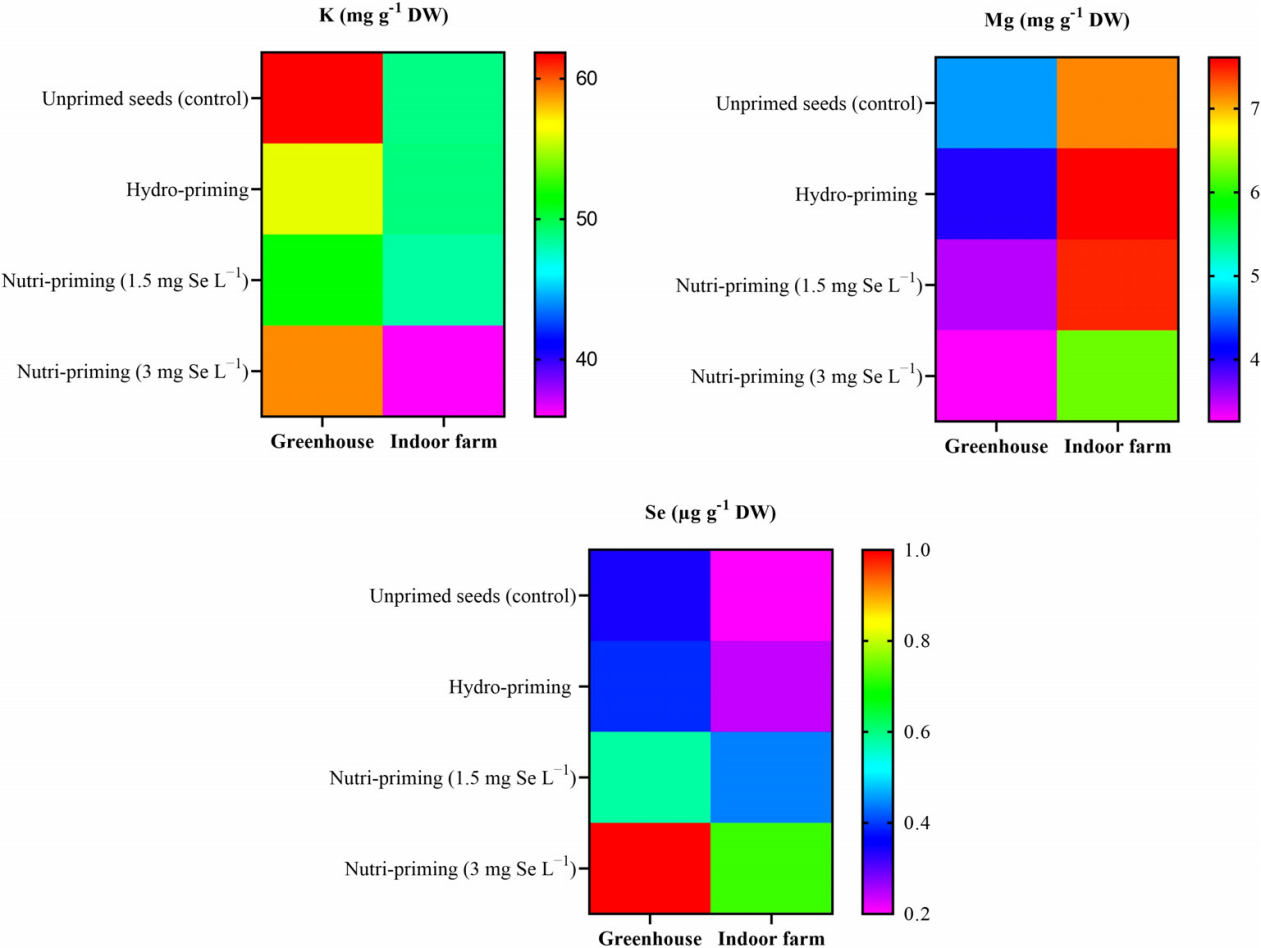
Visual representation of the trends of mineral elements (K, Mg, Se) among treatments as well as between greenhouse and indoor farm conditions.

As a result of the analysis of mineral concentration, to observe the effect of the treatments, there was no difference in the content of K and Mg in the dill microgreens after Se-biofortification through priming, but there was a significant increase in the amount of Se (**Table 3** and **Figure 2**). The Se added to the seeds was absorbed and accumulated in the aerial parts of the microgreens, even if the detected levels were very low. In greenhouse condition, nutri-priming with 3 mg Se L^−1^ significantly increased the Se concentration of microgreens by 2.9 times compared to unprimed seeds. The difference is also significant compared to other treatments. The same conditions were also maintained in the indoor cultivation system, with the difference that the content of Se in microgreens treated with nutri-priming of 3 mg Se L^−1^ was 3.6 times that of unprimed seeds. Also in this case, the treatment showed the highest average value. According to the findings of this study, Se can be enhanced in microgreens through priming to ensure that the level of Se in the plant tissue does not exceed the permissible limit. The t-test results showed that there was a significant difference between the greenhouse and indoor farm systems in terms of K and Mg contents, while the Se content did not show a significant difference between the two systems (**Table 2** and **Figure 2**). Interestingly, the K concentration of microgreens grown indoor was approximately 6 times that of those grown in greenhouse (**Table 2**). 

**Table 3:** Mineral concentration (K, Mg, and Se) of dill microgreens treated with different levels of Se priming in both greenhouse and indoor farm systems.

Cultivation system	Treatments	K (mg g ^-1^ DW)	Mg (mg g ^-1^ DW)	Se (µg g ^-1^ DW)
Greenhouse	Unprimed seeds (control)	61.85± 2.89	4.65 ± 0.20	0.34 ± 0.01 c
	Hydro-priming	56.03 ± 3.61	3.99 ± 0.21	0.39 ± 0.06 c
	Nutri-priming (1.5 mg Se L^−1^)	51.32 ± 1.79	3.51 ± 0.17	0.58 ± 0.01 b
	Nutri-priming (3 mg Se L^−1^)	59.03 ± 16.26	3.26 ± 1.11	1 ± 0.07 a
Significance		ns	ns	***
Indoor farm	Unprimed seeds (control)	48.79 ± 3.40	7.15 ± 0.13	0.20 ± 0.005 c
	Hydro-priming	49 ± 1.27	7.61 ± 0.09	0.24 ± 0.01 bc
	Nutri-priming (1.5 mg Se L^−1^)	48.20 ± 3.96	7.46 ± 0.24	0.44 ± 0.04 b
	Nutri-priming (3 mg Se L^−1^)	35.94 ± 5.20	6.24 ± 0.69	0.72 ± 0.13 a
Significance		ns	ns	**

**, ***, and ns indicate significant at p ≤ 0.01, 0.001, and non-significant, respectively. All data are expressed as mean ± standard error (SE), n=3. The least significant difference test (LSD) was used to compare the means of treatments for each trait (p=0.05). Different letters in each column, where present, indicate significant differences.

### 3.3 Color values

In the greenhouse system, dill leaves did not respond significantly to the color characteristics as a function of different Se-priming treatments (**Table 4**). This trend was the same for microgreens grown in indoor conditions, although the a* value was significantly different among the treatments (p ≤ 0.05). The nutri-priming with 3 mg Se L^−1^ in greenhouse conditions and the nutri-priming with 1.5 mg Se L^−1^ in indoor farm conditions were most effective in increasing the value of greenness (−a*), and in the last one in a significantly way than unprimed seeds. Yellowness (b*), leaf saturation/vivid color (Chroma), and hue of dill leaves were not affected by Se-priming treatments. Based on t-test results, it was observed that L*, a*, b*, and Chroma of microgreens grown in the indoor farm system were significantly higher than those grown in the greenhouse system (**Table 2**). The differences were visible by observing directly the plants: in indoor, they appeared to be of a more intense green color, a positive characteristic for the visual appearance of the product, which is more attractive to the consumer.

**Table 4:** Canopy colorimetric indices of dill microgreens treated with different levels of priming in both greenhouse and indoor farm system conditions.

Cultivation system	Treatments	L*	a*	b*	Chroma	Hue
Greenhouse	Unprimed seeds (control)	24.43 ± 2.23	−4.65 ± 1.82	16.56 ± 1.89	17.32 ± 2.27	75.38 ± 4.22
	Hydro-priming	26.61 ± 2.61	−6.21 ± 1.15	18.10 ± 2.26	19.16 ± 2.50	71.31 ± 1.04
	Nutri-priming (1.5 mg Se L^−1^)	23.03 ± 1.71	−4.23 ± 0.60	15.67 ± 0.76	16.29 ± 0.86	75.02 ± 1.33
	Nutri-priming (3 mg Se L^−1^)	31.29 ± 2.20	−7.38 ± 1.32	19.03 ± 1.37	20.45 ± 1.74	69.17 ± 2.29
Significance		ns	ns	ns	ns	ns
Indoor farm	Unprimed seeds (control)	28.25 ± 1.75	−7.10 ± 0.36 a	19.30 ± 0.76	20.61 ± 0.75	69.77 ± 1.13
	Hydro-priming	30.86 ± 2.08	−8.20 ± 0.73 ab	22.04 ± 1.46	23.54 ± 1.57	69.62 ± 1.05
	Nutri-priming (1.5 mg Se L^−1^)	33.73 ± 0.69	−9.72 ± 0.47 b	23.46 ± 0.81	25.40 ± 0.92	67.51 ± 0.43
	Nutri-priming (3 mg Se L^−1^)	30.65 ± 0.52	−8.07 ± 0.48 ab	21.95 ± 0.88	23.41 ± 0.96	69.82 ± 0.66
Significance		ns	*	ns	ns	ns

*, and ns indicate significant at p ≤ 0.05 and non-significant, respectively. All data are expressed as mean ± standard error (SE), n=3. The least significant difference test (LSD) was used to compare the means of treatments for each trait (p=0.05). Different letters in each column, where present, indicate significant differences.

### 3.4 Pigments level

In both cultivation systems, the concentration of chlorophyll *a*, chlorophyll *b*, total chlorophylls, and carotenoids were not affected by the treatments. However, different Se-priming treatments significantly affected the chlorophyll *a/b* ratio and the chlorophylls/carotenoids ratio in greenhouse (**Table 5**). More precisely, in greenhouse, nutri-priming with 3 mg Se L^−1^ significantly reduced the chlorophyll *a/b* ratio and the chlorophylls/carotenoids ratio compared to unprimed seeds by 12.91% and 15.78%, respectively. According to the t-test results, there was no significant difference in the concentration of photosynthetic pigments of dill microgreens grown in either system, although the average chlorophyll *a* concentration of the microgreens grown indoor was 19.04% higher than those grown in greenhouse (**Table 2**).

**Table 5:** Photosynthetic pigments of dill microgreens treated with different levels of priming in both greenhouse and indoor farm system.

Cultivation system	Treatments	Chlorophyll *a* (μg mg^-1^ FW)	Chlorophyll *b* (μg mg^-1^ FW)	Total chlorophylls (μg mg^-1^ FW)	Carotenoids (μg mg^-1^ FW)	Chlorophyll *a/b* ratio	Chlorophylls/carotenoids ratio
Greenhouse	Unprimed seeds (control)	0.667 ± 0.04	0.246 ± 0.02	0.914 ± 0.06	0.180 ± 0.00	2.71 ± 0.04 a	0.19 ± 0.00 a
	Hydro-priming	0.646 ± 0.06	0.235 ± 0.01	0.882 ± 0.08	0.167 ± 0.01	2.73 ± 0.04 a	0.18 ± 0.00 a
	Nutri-priming (1.5 mg Se L^−1^)	0.658 ± 0.02	0.234 ± 0.01	0.892 ± 0.03	0.172 ± 0.00	2.82 ± 0.09 a	0.19 ± 0.00 a
	Nutri-priming (3 mg Se L^−1^)	0.577 ± 0.02	0.242 ± 0.00	0.819 ± 0.02	0.137 ± 0.00	2.38 ± 0.10 b	0.16 ± 0.00 b
Significance		ns	ns	ns	ns	*	***
Indoor farm	Unprimed seeds (control)	0.600 ± 0.04	0.221 ± 0.01	0.822 ± 0.05	0.158 ± 0.01	2.71 ± 0.10	0.19 ± 0.00
	Hydro-priming	0.494 ± 0.02	0.198 ± 0.01	0.693 ± 0.04	0.128 ± 0.00	2.50 ± 0.05	0.18 ± 0.00
	Nutri-priming (1.5 mg Se L^−1^)	0.570 ± 0.09	0.232 ± 0.03	0.802 ± 0.12	0.146 ± 0.02	2.43 ± 0.05	0.18 ± 0.00
	Nutri-priming (3 mg Se L^−1^)	0.660 ± 0.02	0.255 ± 0.00	0.915 ± 0.03	0.172 ± 0.00	2.59 ± 0.12	0.18 ± 0.00
Significance		ns	ns	ns	ns	ns	ns

*and ns indicate significant at p ≤ 0.05 and non-significant, respectively. *** represents significant at p≤0.001. All data are expressed as mean ± standard error (SE), n=3. The least significant difference test (LSD) was used to compare the means of treatments for each trait (p=0.05). Different letters in each column, where present, indicate significant differences.

### 3.5 Phenolic index, soluble sugars, nitrate concentration, and °Brix

**Figure 3:**
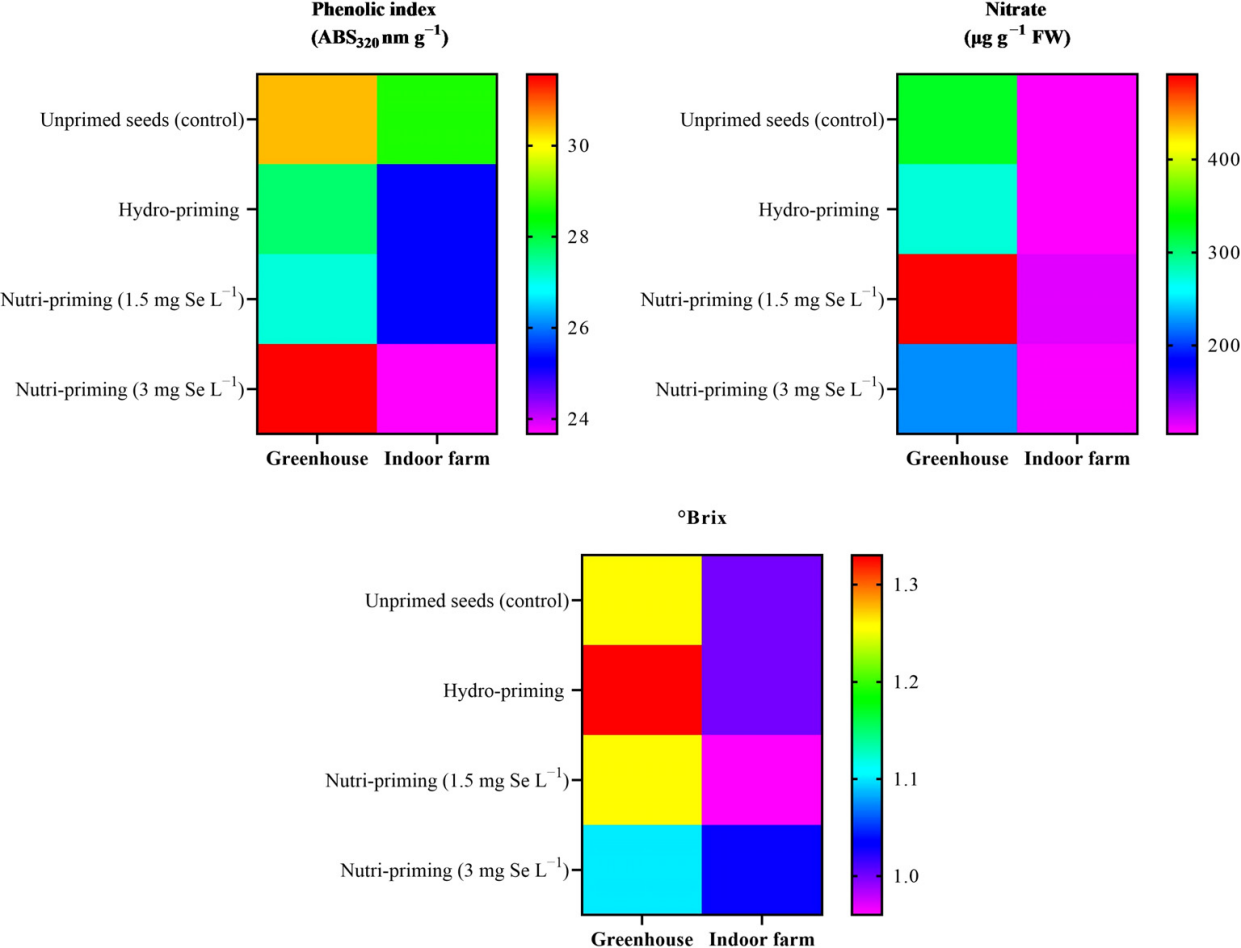
Visual representation of trends in phenolic index, nitrate concentration, and Brix° of dill microgreens among treatments as well as between greenhouse and indoor farm conditions.

Under both the greenhouse and indoor farm system, the results showed that there was no significant effect of the Se-priming on the phenolic index, soluble sugars, and nitrate concentration, except for °Brix, which was affected by different levels of treatments under greenhouse condition, as reported in **Table 6** and in **Figure 3**. In addition, °Brix was not affected by Se-priming treatments under indoor farm system (**Table 6**). In the greenhouse, 3 mg Se L^−1^ caused a decrease in °Brix by 12.69%, 17.29%, and 12.69% compared to unprimed, hydro-priming, and 1.5 mg Se L^−1^, respectively. According to the t-test (**Table 2**), microgreens grown under greenhouse conditions had a higher value of phenolic index than those grown indoor farm. The trend of nitrate content and °Brix of microgreens grown in indoor farm system decreased compared to those grown in greenhouse system by 66.81 and 9.90%, respectively. The results of t-test also showed that there was no significant difference for soluble sugars in both cultivation systems (**Table 2**).

**Table 6:** Phenolic index, soluble sugars, nitrate concentration, and Brix° of dill microgreens treated with different levels of priming in both greenhouse and indoor farm systems.

Cultivation system	Treatments	Phenolic index (ABS_320_nm g^−1^)	Soluble sugars (mg kg^−1^ FW)	Nitrate (μg g^−1^ FW)	°Brix
Greenhouse	Unprimed seeds (control)	30.425 ± 1.86	4893.5 ± 372.1	323.9 ± 57.20	1.26 ± 0.03 a
	Hydro-priming	27.719 ± 3.25	5576.1 ± 75.8	273.1 ± 36.63	1.33 ± 0.03 a
	Nutri-priming (1.5 mg Se L^−1^)	27.096 ± 2.07	4882.4 ± 691.5	491.4 ± 144.89	1.26 ± 0.08 a
	Nutri-priming (3 mg Se L^−1^)	31.563 ± 2.14	4299.7 ± 585.2	224.2 ± 7.54	1.10 ± 0.00 b
Significance		ns	ns	ns	*
Indoor farm	Unprimed seeds (control)	28.623 ± 2.75	5590 ± 177.6	105.52 ± 24.73	1 ± 0.05
	Hydro-priming	25.217 ± 2.78	5148 ± 769.3	104.86 ± 20.83	1 ± 0.05
	Nutri-priming (1.5 mg Se L^−1^)	25.284 ± 2.26	5337 ± 1197.5	115.88 ± 43.01	0.96 ± 0.12
	Nutri-priming (3 mg Se L^−1^)	23.669 ± 1.66	5148 ± 900.5	109.48 ± 27.76	1.03 ± 0.06
Significance		ns	ns	ns	ns

* and ns indicate significant at p ≤ 0.05 and non-significant, respectively. All data are expressed as mean ± standard error (SE), n=3. The least significant difference test (LSD) was used to compare the means of treatments for each trait (p=0.05). Different letters in each column, where present, indicate significant differences.

### 3.6 Principal Component Analysis

**Figure 4:**
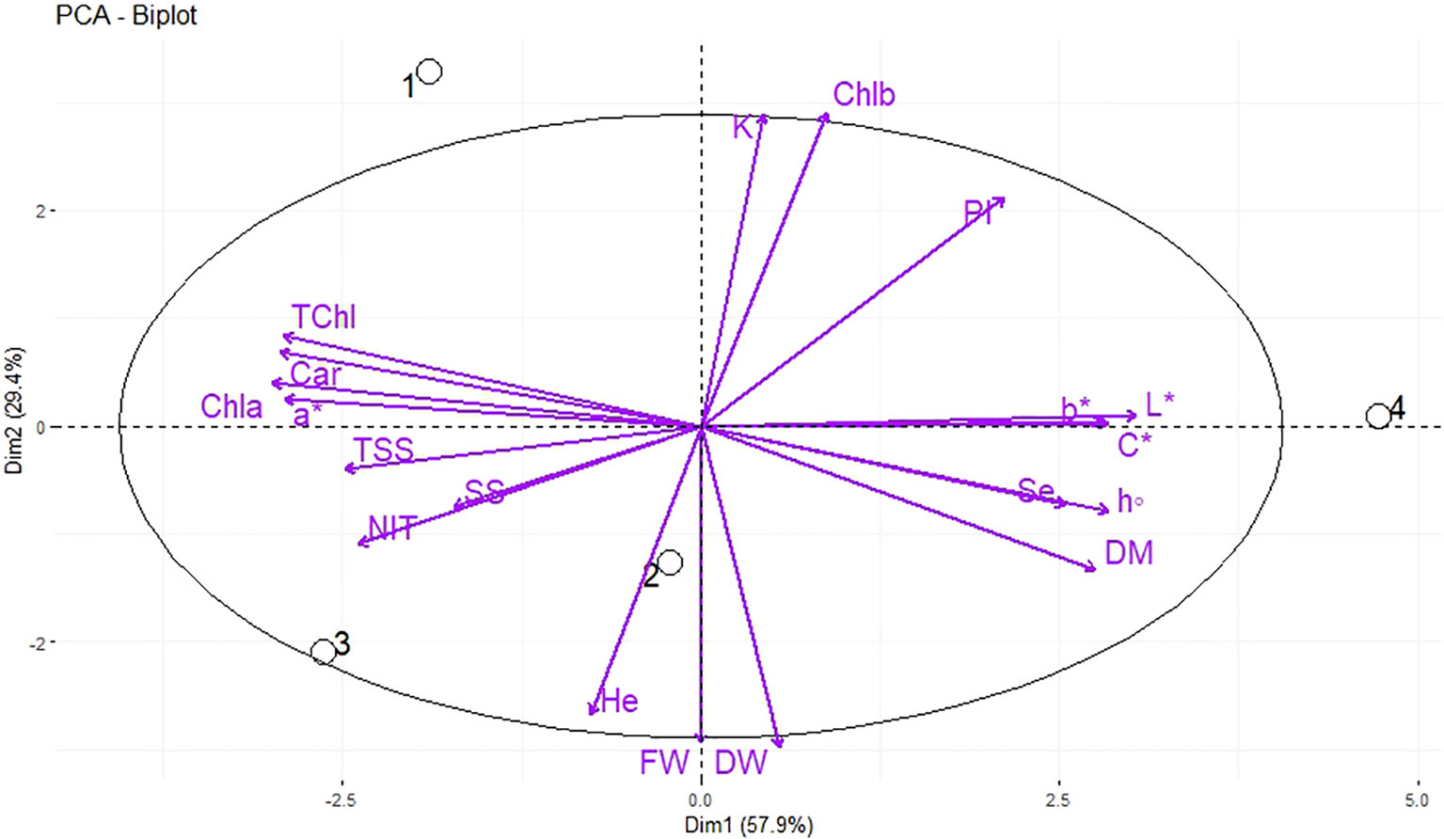
Principal Component Analysis (PCA) of morphological, biochemical, and elements traits of dill microgreens under different levels of Se priming (greenhouse system). The location of the treatments (1, control; 2, Hydro-priming; 3, nutri-priming of 1.5 mg Se L^−1^; 4, nutri-priming of 3 mg Se L^−1^) is presented in the individuals-PCA, and the traits are shown in the variables-PCA. Fresh weight (FW), dry weight (DW), dry matter (DM), height (He), chlorophyll *a* (Chl *a*), *b* (Chl *b*), and total (TChl), carotenoids (Car), a*, b*, L*, chroma (C*), hue (h°), soluble sugars (SS), phenolic index (PI), nitrate (NIT), TSS, potassium (K), magnesium (Mg), selenium (Se).

The PCA was used for the analysis of the morphological and qualitative characteristics of dill microgreens in response to different levels of Se priming, in both greenhouse and indoor farm system, to provide a comprehensive overview and interpretation of the obtained results. Under greenhouse system (E1), the principal component (PC1) accounted for 57.9% of the cumulative variance, while PC2 explained 29.4% of the total variance (**Figure 4**). The nutri-priming of 3 mg Se L^−1^ was positioned on the positive side of PC1 in the upper right quadrant of PCA-Biplot (**Figure 4**), as it produced plants with higher potassium (K), chlorophyll *b* (Chl *b*), phenolic index (PI), b*, chroma (c*), and L*. Moreover, the hydro-priming treatment was located on the negative side of PC2 in the lower left quadrant of PCA-Biplot (**Figure 4**), as it produced plants with higher fresh weight (FW) and dry weight (DW).

**Figure 5:**
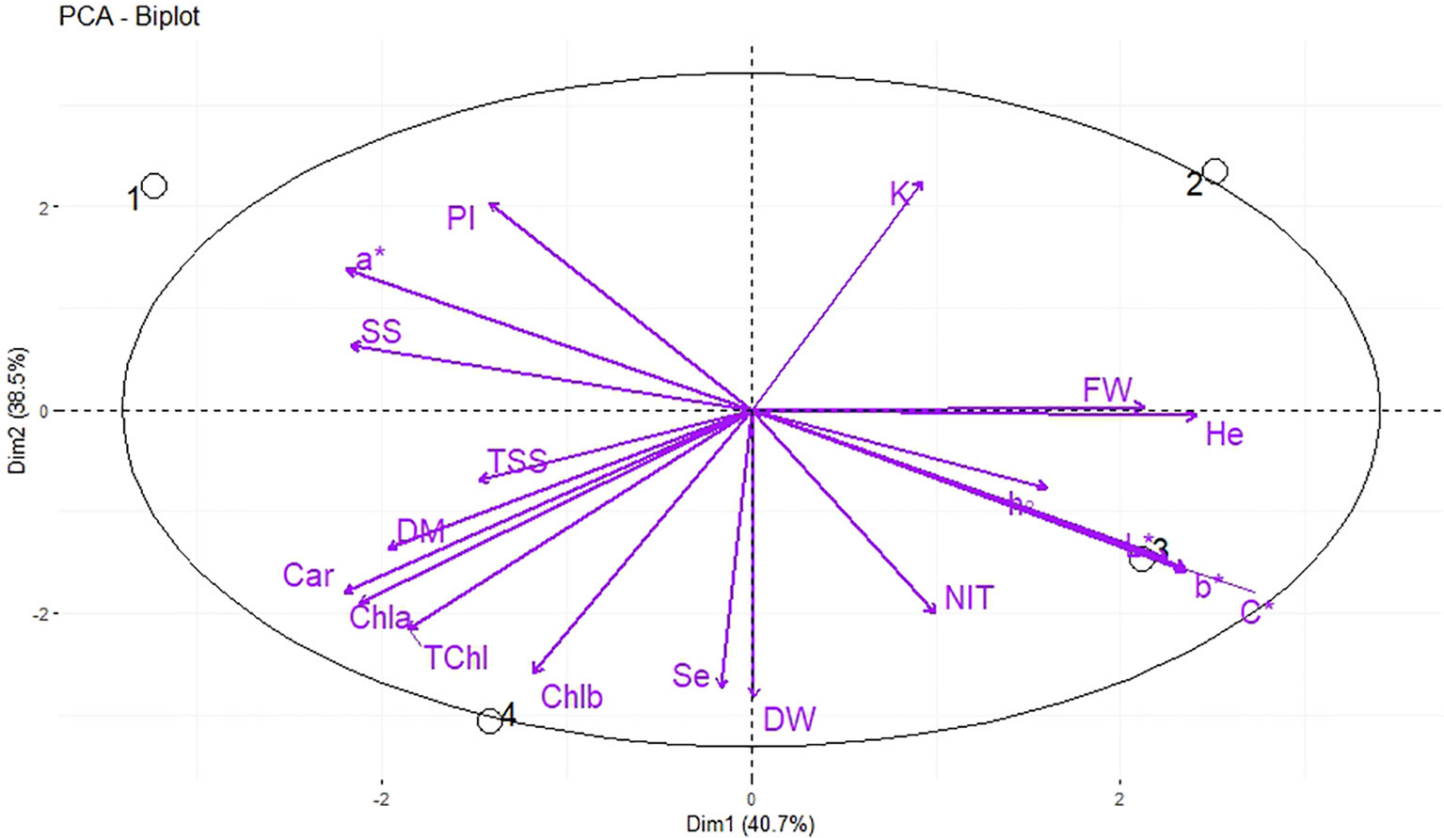
Principal Component Analysis (PCA) of morphological, biochemical, and elements traits of dill microgreens under different levels of Se priming (indoor farm system). The location of the treatments (1, control; 2, Hydro-priming; 3, nutri-priming of 1.5 mg Se L^−1^; 4, nutri-priming of 3 mg Se L^−1^) is presented in the individuals-PCA, and the traits are shown in the variables-PCA. Fresh weight (FW), dry weight (DW), dry matter (DM), height (He), chlorophyll *a* (Chl *a*), *b* (Chl *b*), and total (TChl), carotenoids (Car), a*, b*, L*, chroma (C*), hue (h°), soluble sugars (SS), phenolic index (PI), nitrate (NIT), TSS, potassium (K), magnesium (Mg), selenium (Se).

Under indoor farm system (E2), the PC1 accounted for 40.7% of the cumulative variance, while PC2 explained 38.5% of the total variance (**Figure 5**). Hydro-priming was placed in the positive side of PC1 in the upper right quadrant of PCA-Biplot (**Figure 5**), as it produced plants with higher K and FW. The nutri-priming of 3 mg Se L^−1^ was located on the negative side of PC2 in the lower left quadrant of PCA-Biplot (**Figure 5**), as it produced plants with higher photosynthetic pigments and Se.

### 3.7 Correlation analysis

**Figure 6:**
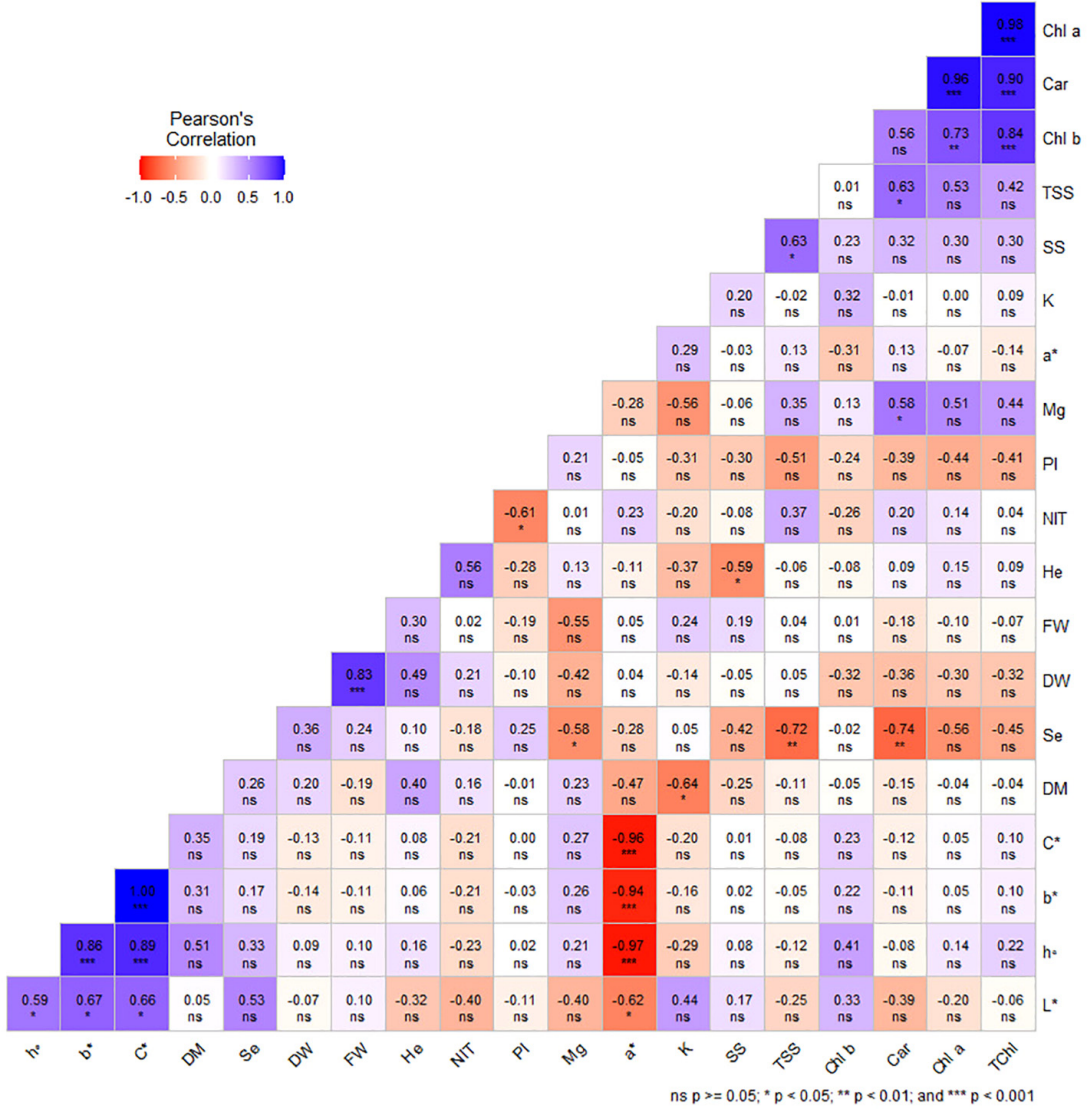
Pearson’s correlation coefficients among studied traits in dill microgreens under different levels of Se priming (greenhouse system). Fresh weight (FW), dry weight (DW), dry matter (DM), height (He), chlorophyll *a* (Chl *a*), *b* (Chl *b*), and total (TChl), carotenoids (Car), a*, b*, L*, chroma (C*), hue (h°), soluble sugars (SS), phenolic index (PI), nitrate (NIT), TSS, potassium (K), magnesium (Mg), selenium (Se).

Under greenhouse system (E1), a* was negatively and significantly correlated with b*, L*, h°, and C*. There was a significant negative correlation between K and DM, as well as a significant negative correlation between Se and Mg, Car, and TSS (°Brix). In addition, it was found that NIT has a significant negative correlation with phenolic index (PI). It has been observed that Car had a significant positive correlation with Mg and TSS. Moreover, a similar relationship was recorded between SS and TSS (**Figure 6**).

**Figure 7:**
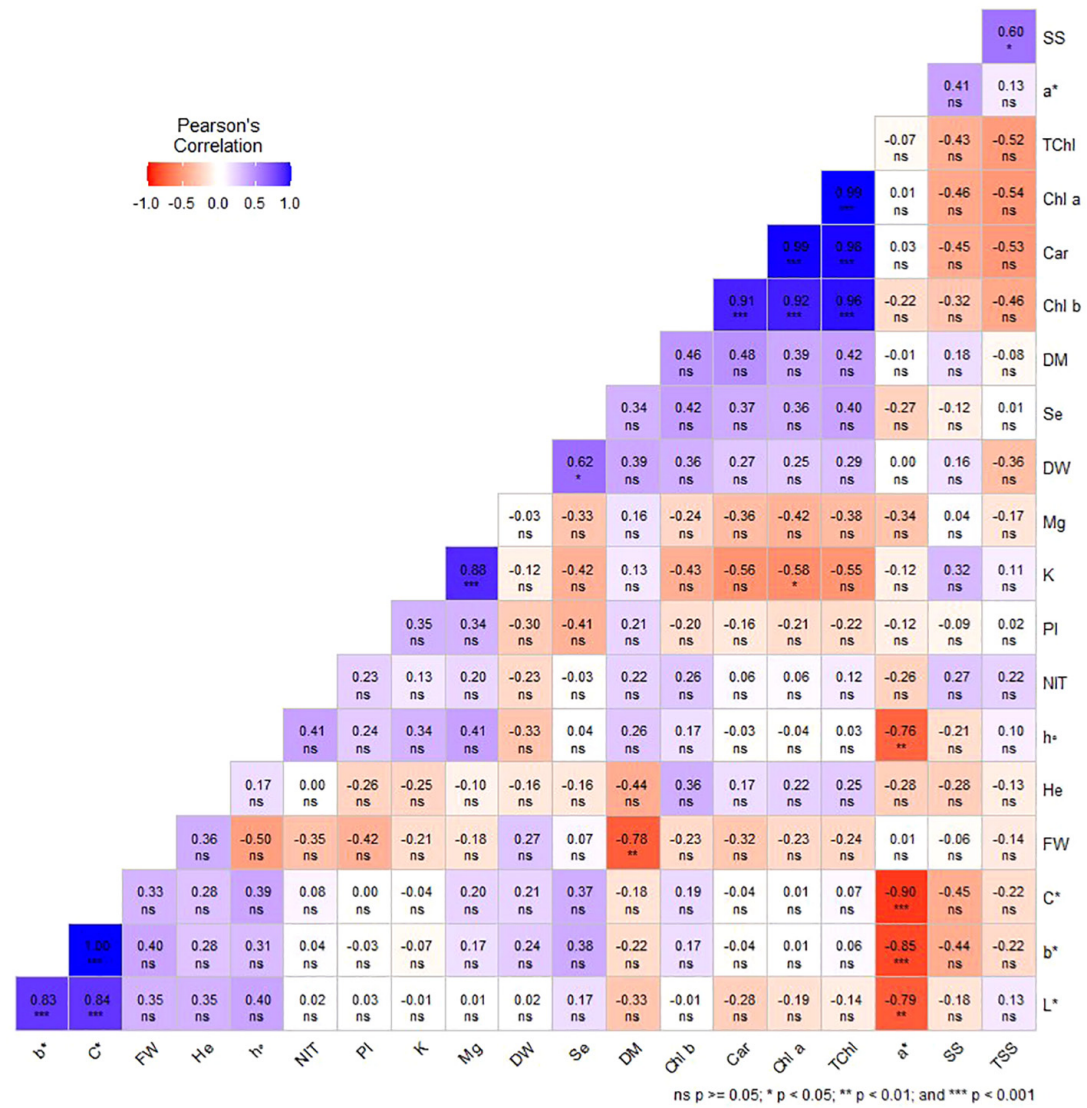
Pearson’s correlation coefficients among studied traits in dill microgreens under different levels of Se priming (indoor farm system). Fresh weight (FW), dry weight (DW), dry matter (DM), height (He), chlorophyll *a* (Chl *a*), *b* (Chl *b*), and total (TChl), carotenoids (Car), a*, b*, L*, chroma (C*), hue (h°), soluble sugars (SS), phenolic index (PI), nitrate (NIT), TSS, potassium (K), magnesium (Mg), selenium (Se).

Under indoor farm system (E2), it was found that FW and DM, as well as K and Chl *a*, were significantly negatively correlated. The results showed that a* was negatively and significantly correlated with b*, L*, h°, and C*. Additionally, K and Mg, DW and Se, and SS and TSS demonstrated significant positive correlations in this study (**Figure 7**).

## 4 Discussion

The aim of enriching crops with essential micronutrients, such as Se, can certainly have positive implications for human health and increase the quality of vegetables. In addition to the final quality of products, the maintenance of good production levels remains of crucial importance. Regarding the plant growth, in our study, it can be observed that the seeds priming improved the microgreens biomass, in terms of FW and yield, even if not in a statistically relevant way. A similar trend can also be observed in the DW data, in which we notice an even more pronounced effect in indoor conditions with Se priming. In literature, it has been reported that Se had no effect on the yield of microgreens of green and purple basil (Pannico et al., 2020), which is consistent with the outcome of this experiment. Other studies have also reported a non-significant effect of Se on microgreens (Puccinelli et al., 2019) and mature plants yield (Skrypnik et al., 2019), confirming this result. Generally, the priming of seeds is well known to enhance the seeds germination, the growth of seedlings, the establishment of plants, and the yield of crops when used in an appropriate manner (Ebert, 2022). It is believed that priming has a beneficial impact on plant growth. This could be because it improves the nutrient use efficiency which allows a higher relative growth rate and it improves the regulation of the water status of the plant, at the same time (Lutts et al., 2016). A greater growth rate of seedlings produced by primed seeds may also be attributed directly to regulating cell cycle and elongation processes through pretreatment (Chen and Arora, 2013; Sung et al., 2008).

It is also interesting to note how the height of microgreens was influenced by the cultivation system: in indoor condition we notice double the heights than the greenhouse condition. There was an acceleration of the growing cycle, which could have allowed the indoor microgreens to be harvested earlier. For experimental reasons, it was decided to harvest dill at the same time in both cultivation systems, but this is certainly an interesting result from the producers’ point of view. In fact, this allows getting to the market sooner with the product and also save inputs. Light intensity and photoperiod are limiting factors for greenhouse production during winter and early autumn due to reduced daily light integrals, which negatively affect plant growth and morphology (Hernández and Kubota, 2014). Since vertical farming provides unlimited opportunities to control the intensity and duration of light, it can effectively address the issue of limited light integrals during wintertime (Voutsinos et al., 2021). Due to this, the good growth of dill microgreens in indoor farm conditions may have been partially due to the presence of adequate light, supplied through LED lamps. In the greenhouse, we only had natural light as a source of lighting.

Regarding the mineral concentration, in our study, it was found that the seeds primed with Se did not change the Mg and K contents. Possibly, the reason for this might be that there was not enough Se exposure in the treatment for dill to trigger the change in Mg and K concentrations. Our results are consistent with that of Abdalla et al. (2022), who reported that the Mg content of lettuce leaves was not altered by Se biofortification, however, the amount of K decreased with an increase in Se. There was an increase in Se level in dill microgreens following seed priming with Se, especially when the concentration was 3 mg Se L^−1^. A significant increase in the level of Se was also observed in wheat (Islam et al., 2020), coriander, green basil, purple basil, and tatsoi (Pannico et al., 2020) microgreens that were biofortified with Se. The results of the current study showed that dill microgreens can be enriched with Se through seed priming. It was found that the Se content in dill, in greenhouse, was 0.58 and 1 µg g^−1^ DW for treatments 1.5 and 3 mg Se L^−1^, respectively, and in indoor farm conditions, the Se content was 0.44 and 0.72 µg g^−1^ DW. The mineral Se has long been recognized as one of the most essential trace minerals for the human body. Its deficiency leads to serious diseases, like cardiovascular disease, cancer, viral infection, and diabetes, among others (Pannico et al., 2020). Supplementing the diet with Se-enriched microgreens, as demonstrated in this study, may effectively address daily Se requirements. This research underscores a promising methodology for enhancing the nutritional profile of dill microgreens, which could have significant implications for both agricultural practices and nutritional strategies. The findings suggest that employing this technique could contribute to improved dietary intake of Se, thereby supporting public health initiatives.

Moving to the other important parameter considered in the trial to define the quality of vegetables, the color and therefore the pigments are crucial because they contribute to the visual appearance of the product (Ferrante et al., 2004). Generally, Se application enhances the biosynthesis of photosynthetic pigments in plants; however, an opposite effect was found for example in rice sprouts by D’Amato et al. (2018) and in coriander microgreens by Pannico et al. (2020), in which Se applications entailed a reduction in the total carotenoids. These findings might denote a possible species-dependent response. In our samples, it has been observed that the Se treatments increased the results of the greenness (a*), more markedly in the indoor farm system, where the observed differences were statistically relevant. It is essential that consumers consider the product’s external appearance when evaluating its quality and making purchase decisions (Trod et al., 2023). A major deterioration symptom of the plant is the loss of green color, caused by the catabolism of chlorophyll, resulting in yellow coloration or yellowing of the plant (Büchert et al., 2011; Muñoz et al., 2021). An explanation for this yellowing can be found in the disassembly of chloroplasts (Fukasawa et al., 2010). It was also observed in a study carried out in broccoli (*Brassica oleracea* L. var. *italica* Plenck) that treatment with Se has been shown to prevent yellowing and increase greenness (Trod et al., 2023). Additionally, Se decline ethylene biosynthesis (Puccinelli et al., 2017), which can explain microgreens’ greenness as well. In plants, chlorophylls play a role in photosynthesis, and are also important as phytonutrients. There is ample evidence that chlorophylls prevent the development of neurodegenerative diseases in humans and have antioxidative, anticarcinogenic, and antimutagenic properties. In our study, Se did not stimulate photosynthetic pigment accumulation, although chlorophyll *a*/*b* ratio and chlorophylls/carotenoids ratio declined under 3 mg Se L^−1^, in greenhouse system. It is unclear whether Se affects chlorophyll accumulation in green leafy vegetables or not. According to a study on spinach (Saffaryazdi et al., 2012), using low Se concentrations resulted in higher chlorophyll *a*, chlorophyll *b*, and total chlorophyll levels. In contrast, in a study conducted by Malorgio et al. (2009), the presence of Se in lettuce and chicory leaves did not have a significant effect on the amount of chlorophylls. In line with our results, neither basil (Puccinelli et al., 2020) nor kale (Lefsrud et al., 2006) leaves showed any significant changes in carotenoid concentrations after applying Se.

Phenolic index, soluble sugars, and nitrate concentration were not affected by different Se-priming treatments. Khaliq et al. (2015) also did not observe a difference in the total phenolics of Se-primed seeds compared to the control in rice seedlings. On the other hand, it was reported that hydro-priming on Indian mustard (*Brassica juncea* L.) seedlings resulted in more phenolics than seedlings that were not hydro-primed (Srivastava et al., 2010). It is well known that phenolic compounds play a protective role in plants, and under various biotic and abiotic stresses, their synthesis and accumulation increase (Khaliq et al., 2015). It is possible that the dill microgreens were not stressed until the Se concentration reached a 3 mg Se L^−1^, which was not unexpected. Se is involved in increasing the accumulation of soluble sugars (Gao et al., 2021), but in our study no increase in soluble sugars was observed. Regarding nitrate, there is presently a lack of understanding of the effects of Se on nitrogen metabolism. It has been found that Se treatment reduced nitrate levels in lettuce plants (Rios et al., 2010), while it had no effect on chicory (Malorgio et al., 2009), spinach (Ferrarese et al., 2012), or chard (Hernández-Castro et al., 2015) plants. There was a reduction in nitrate in dill microgreens under indoor farm conditions compared to greenhouse conditions, which may be related to the effect of light (better light quality, as seen above for other determinations) on increasing the activity of nitrogen metabolism related enzymes. This interesting effect will certainly need to be investigated, although nitrate accumulation is not an issue in microgreens as they generally have lower levels than adult vegetables. In greenhouse conditions, °Brix decreased in a significant way in the presence of 3 mg Se L^−1^. This effect, to be evaluated in more detail in the future, could be linked to a perturbation of the treatment on the photosynthetic process, albeit slight. In fact, we noted decreases, although not significant, in the average values of Chl *a*, total Chl, Car, and soluble sugars, and, moreover, a significant reduction in the chlorophyll *a*/*b* ratio and chlorophylls/carotenoids ratio, as previously reported. These parameters are all closely related to photosynthesis and to the production of carbon skeletons (sugars). It is also important to highlight that a good availability of sugars is useful for promoting the assimilation of nitrate in vegetables.

## 5 Conclusions

The present research was conducted to evaluate the effects of different levels of Se biofortification, through seed priming, on the growth and quality of dill microgreens cultivated in two different environments (greenhouse and indoor farm). The results showed that the production of microgreens from Se-enriched seeds could be a good technique for obtaining microgreens with a high nutritional value. Sodium selenate affected, in both tested conditions, the concentration of Se in dill, confirming the effectiveness of the treatment, and other analyzed parameters, like the color, chlorophyll *a*/*b* ratio, chlorophylls/carotenoids ratio, and Brix°, with different trends in some cases between greenhouse and indoor farm conditions. The findings of this study also showed that chlorophylls and carotenoids concentration, phenolic index, soluble sugars, and nitrate content of dill microgreens are not affected by different Se-priming treatments. From a general view of the results, the concentration 3 mg Se L^−1^ seems to be the most promising, but it could be useful to test other concentrations as well (above 3 mg Se L^−1^). The cultivation environment influenced the outcomes in a different way: indoor farming looks like a promising system to increase the production and the commercial value (quality) of the product. The more marked positive effect was observed on the morphological traits studied. It is also worth mentioning the interesting effect on the acceleration in the growth of microgreens.

The results confirm that sodium selenate biofortification of microgreens can allow producing functional foods by increasing the Se content, with additional benefits for human health. Microgreens are confirmed as valid supplement to the diet to help meet the needs of the daily requirement of Se. In future research, it is suggested that higher concentrations of Se should be used in biofortification of vegetable products through seed priming in order to achieve optimum results.

## Data Availability

The raw data supporting the conclusions of this article will be made available by the authors, without undue reservation.

## References

[B1] Abdalla M. A. Lentz C. Mühling K. H. (2022). Crosstalk between selenium and sulfur is associated with changes in primary metabolism in lettuce plants grown under se and S enrichment. Plants 11 (7), 927. doi: 10.3390/plants11070927 35406907 PMC9002494

[B2] Amitrano C. Paglialunga G. Battistelli A. De Micco V. Del Bianco M. Liuzzi G.. (2023). Defining growth requirements of microgreens in space cultivation via biomass production, morpho-anatomical and nutritional traits analysis. Front. Plant Sci. 14. doi: 10.3389/fpls.2023.1190945 PMC10394706 37538067

[B3] Bhaswant M. Shanmugam D. K. Miyazawa T. Abe C. Miyazawa T. (2023). Microgreens—A comprehensive review of bioactive molecules and health benefits. Molec. 28, 867. doi: 10.3390/molecules28020867 PMC9864543 36677933

[B4] Bhatia P. Gupta M. (2022). Micronutrient seed priming: new insights in ameliorating heavy metal stress. Environ. Sci. pollut. Res. 29, 58590–58606. doi: 10.1007/s11356-022-21795-6 35781664

[B5] Brazaitytė A. Miliauskienė J. Vaštakaitė-Kairienė V. Sutulienė R. Laužikė K. Duchovskis P.. (2021). Effect of different ratios of blue and red led light on brassicaceae microgreens under a controlled environment. Plants 10, 801. doi: 10.3390/plants10040801 33921895 PMC8073284

[B6] Büchert A. M. Civello P. M. Martínez G. A. (2011). Chlorophyllase versus pheophytinase as candidates for chlorophyll dephytilation during senescence of broccoli. J. Plant Physiol. 168, 337–343. doi: 10.1016/j.jplph.2010.07.011 20727617

[B7] Bulgari R. Negri M. Santoro P. Ferrante A. (2021). Quality evaluation of indoor-grown microgreens cultivated on three different substrates. Horticulturae 7 (5), 96. doi: 10.3390/horticulturae7050096

[B8] Bulgari R. Sheikhi H. Ertani A. Delshad M. Nicola S. (2022). “Growing biofortified microgreens in indoor farm and in greenhouse: a comparison,” in XXXI international horticultural congress (IHC2022): international symposium on advances in vertical farming 1369, 93–100. doi: 10.17660/ActaHortic.2023.1369.11

[B9] Cataldo D. A. Haroon M. Sehrader L. E. Youngs V. L. (1975). Rapid colorimetric determination of nitrate in plant tissue by titration of salicylic acid. Commun. Soil Sci. Plant Anal. 6, 71–80. doi: 10.1080/00103627509366547

[B10] Chen K. Arora R. (2013). Priming memory invokes seed stress-tolerance. Env. Exp. Bot. 94, 33–45. doi: 10.1016/j.envexpbot.2012.03.005

[B11] Ciriello M. Formisano L. El-Nakhel C. Zarrelli A. Giordano M. De Pascale S.. (2023). Iodine biofortification of four microgreens species and its implications for mineral composition and potential contribution to the recommended dietary intake of iodine. Sci. Hortic. 320, 112229. doi: 10.1016/j.scienta.2023.112229

[B12] D’Amato R. Fontanella M. C. Falcinelli B. Beone G. M. Bravi E. Marconi O.. (2018). Selenium biofortification in rice (*Oryza sativa* L.) sprouting: effects on Se yield and nutritional traits with focus on phenolic acid profile. J. Agr. Food Chem. 66, 4082–4090. doi: 10.1021/acs.jafc.8b00127 29619819

[B13] Ebert A. W. (2022). Sprouts and microgreens—Novel food sources for healthy diets. Plants 11 (4), 571. doi: 10.3390/plants11040571 35214902 PMC8877763

[B14] Ferrante A. Incrocci L. Maggini R. Serra G. Tognoni F. (2004). Colour changes of fresh-cut leafy vegetables during storage. J. Food. Agr. Env. 2, 40–44.

[B15] Ferrarese M. Sourestani M. Quattrini E. Schiavi M. Ferrante A. (2012). Biofortification of spinach plants applying selenium in the nutrient solution of floating system. Veg. Crop Res. Bull. 76, 127–136. doi: 10.2478/v10032-012-0009-y

[B16] Fukasawa A. Suzuki Y. Terai H. Yamauchi N. (2010). Effects of postharvest ethanol vapor treatment on activities and gene expression of chlorophyll catabolic enzymes in broccoli florets. Postharvest Biol. Technol. 55, 97–102. doi: 10.1016/j.postharvbio.2009.08.010

[B17] Gao M. He R. Shi R. Li Y. Song S. Zhang Y.. (2021). Combination of selenium and UVA radiation affects growth and phytochemicals of broccoli microgreens. Molec. 26 (15), 4646. doi: 10.3390/molecules26154646 PMC8348033 34361799

[B18] Guardiola-Márquez C. E. García-Sánchez C. V. Sánchez-Arellano Ó.A. Bojorquez-Rodríguez E. M. Jacobo-Velázquez D. A. (2023). Biofortification of Broccoli Microgreens (*Brassica oleracea* var. italica) with Glucosinolates, Zinc, and Iron through the Combined Application of Bio-and Nanofertilizers. Foods 12, 3826. doi: 10.3390/foods12203826 37893719 PMC10606838

[B19] Hernández R. Kubota C. (2014). Growth and morphological response of cucumber seedlings to supplemental red and blue photon flux ratios under varied solar daily light integrals. Sci. Hortic. 173, 92–99. doi: 10.1016/j.scienta.2014.04.035

[B20] Hernández-Castro E. Trejo-Téllez L. I. Gómez-Merino F. C. Rodríguez-Mendoza M. N. Sánchez-García P. Robledo-Paz A. (2015). Bioaccumulation of iron, selenium, nitrate, and proteins in chard shoots. J. Soil Sci. Plant Nutr. 15, 694–710. doi: 10.4067/S0718-95162015005000047

[B21] Islam M. Z. Park B. J. Kang H. M. Lee Y. T. (2020). Influence of selenium biofortification on the bioactive compounds and antioxidant activity of wheat microgreen extract. Food Chem. 309. doi: 10.1016/j.foodchem.2019.125763 31787393

[B22] Izydorczyk G. Ligas B. Mikula K. Witek-Krowiak A. Moustakas K. Chojnacka K. (2021). Biofortification of edible plants with selenium and iodine – A systematic literature review. Sci. Total Environ. 754, 141983. doi: 10.1016/j.scitotenv.2020.141983 33254892

[B23] Ke D. Saltveit M. E. Jr. (1989). Wound-induced ethylene production, phenolic metabolism and susceptibility to russet spotting in iceberg lettuce. Physiol. Plant 76, 412–418. doi: 10.1111/j.1399-3054.1989.tb06212.x

[B24] Khaliq A. Aslam F. Matloob A. Hussain S. Geng M. Wahid A.. (2015). Seed priming with selenium: Consequences for emergence, seedling growth, and biochemical attributes of rice. Biol. Trace Elem. Res. 166, 236–244. doi: 10.1007/s12011-015-0260-4 25690516

[B25] Kyriacou M. C. El-Nakhel C. Pannico A. Graziani G. Zarrelli A. Soteriou G. A.. (2021). Ontogenetic variation in the mineral, phytochemical and yield attributes of brassicaceous microgreens. Foods 10, 1032. doi: 10.3390/foods10051032 34068729 PMC8151805

[B26] Lefsrud M. G. Kopsell D. A. Kopsell D. E. Randle W. M. (2006). Kale carotenoids are unaffected by, whereas biomass production, elemental concentrations, and selenium accumulation respond to, changes in selenium fertility. J. Agric. Food Chem. 54, 1764–1771. doi: 10.1021/jf052478y 16506831

[B27] Lenzi A. Orlandini A. Bulgari R. Ferrante A. Bruschi P. (2019). Antioxidant and mineral composition of three wild leafy species: A comparison between microgreens and baby greens. Foods 8 (10), 487. doi: 10.3390/foods8100487 31614816 PMC6835962

[B28] Lichtenthaler H. K. (1987). Chlorophylls and carotenoids: pigments of photosynthetic biomembranes, in. Methods Enzymol. 148, 350–382. doi: 10.1016/0076-6879(87)48036-1

[B29] Lone J. K. Pandey R. (2024). Microgreens on the rise: Expanding our horizons from farm to fork. Heliyon 10 (4), e25870. doi: 10.1016/j.heliyon.2024.e25870 38887378 PMC11180960

[B30] Lutts S. Benincasa P. Wojtyla L. Kubala S. Pace R. Lechowska K.. (2016). Seed priming: new comprehensive approaches for an old empirical technique. New Challenges Seed Biol. - Basic Transl. Res. Driv. Seed Technol., 1–46. doi: 10.5772/64420

[B31] Malorgio F. Diaz K. E. Ferrante A. Mensuali-Sodi A. Pezzarossa B. (2009). Effects of selenium addition on minimally processed leafy vegetables grown in a floating system. J. Sci. Food Agric. 89, 2243–2251. doi: 10.1002/jsfa.3714

[B32] McGuire R. G. (1992). Reporting of objective color measurements. HortScience 27 (12), 1254–1255. doi: 10.21273/hortsci.27.12.1254

[B33] Mezeyová I. Hegedűsová A. Golian M. Andrejiová A. Šlosár M. Mezey J. (2022 1096). Influence of microgreens biofortification with selenium on their quantitative and qualitative parameters. Agronomy 12 (5), 1096. doi: 10.3390/agronomy12051096

[B34] Muñoz F. F. Stoffel M. M. Céccoli G. Trod B. S. Daurelio L. D. Bouzo C. A.. (2021). Improving the foliar biofortification of broccoli with selenium without commercial quality losses. Crop Sci. 61, 4218–4228. doi: 10.1002/csc2.20630

[B35] Newman R. G. Moon Y. Sams C. E. Tou J. C. Waterland N. L. (2021). Biofortification of sodium selenate improves dietary mineral contents and antioxidant capacity of culinary herb microgreens. Front. Plant Sci. 12. doi: 10.3389/fpls.2021.716437 PMC8375293 34421969

[B36] Pannico A. El-Nakhel C. Graziani G. Kyriacou M. C. Giordano M. Soteriou G. A.. (2020). Selenium biofortification impacts the nutritive value, polyphenolic content, and bioactive constitution of variable microgreens genotypes. Antioxidants 9, 272. doi: 10.3390/antiox9040272 32218153 PMC7222195

[B37] Poudel P. Di Gioia F. Lambert J. D. Connolly E. L. (2023). Zinc biofortification through seed nutri-priming using alternative zinc sources and concentration levels in pea and sunflower microgreens. Front. Plant Sci. 14. doi: 10.3389/fpls.2023.1177844 PMC10150129 37139105

[B38] Puccinelli M. Malorgio F. Pezzarossa B. (2017). Selenium enrichment of horticultural crops. Molecules 22, 1–18. doi: 10.3390/molecules22060933 PMC6152644 28587216

[B39] Puccinelli M. Malorgio F. Rosellini I. Pezzarossa B. (2019). Production of selenium-biofortified microgreens from selenium-enriched seeds of basil. J. Sci. Food Agric. 99, 5601–5605. doi: 10.1002/jsfa.9826 31149731

[B40] Puccinelli M. Pezzarossa B. Pintimalli L. Malorgio F. (2021). Selenium biofortification of three wild species, *Rumex acetosa* L., *Plantago coronopus* L., and *Portulaca oleracea* L., grown as microgreens. Agronomy 11 (6), 1155. doi: 10.3390/agronomy11061155

[B41] Puccinelli M. Pezzarossa B. Rosellini I. Malorgio F. (2020). Selenium enrichment enhances the quality and shelf life of basil leaves. Plants 9, 801. doi: 10.3390/plants9060801 32604830 PMC7355943

[B42] R Core Team (2020). “Development core team 2020,” in A language and environment for statistical computing. R found. Stat. Comput, vol. 3. (Vienna, Austria). Available at: https://www.R-project.org.

[B43] Rios J. J. Blasco B. Rosales M. A. Sanchez-Rodriguez E. Leyva R. Cervilla L. M.. (2010). Response of nitrogen metabolism in lettuce plants subjected to different doses and forms of selenium. J. Sci. Food Agric. 90, 1914–1919. doi: 10.1002/jsfa.4032 20602511

[B44] Saffaryazdi A. Lahouti M. Ganjeali A. Bayat H. (2012). Impact of selenium supplementation on growth and selenium accumulation on spinach (*Spinacia oleracea* L.) plants. Not. Sci. Biol. 4, 95–100. doi: 10.15835/nsb448029

[B45] Shahverdi M. A. Omidi H. Tabatabaei S. J. (2017). Efeito do amortecimento de nutrientes nos índices de germinação e nas características fisiológicas da plântula de stevia sob o estresse de salinidade. J. Seed Sci. 39, 353–362. doi: 10.1590/2317-1545v39n4172539

[B46] Skrypnik L. Novikova A. Tokupova E. (2019). Improvement of phenolic compounds, essential oil content and antioxidant properties of sweet basil (*Ocimum basilicum* L.) depending on type and concentration of selenium application. Plants 8, 458. doi: 10.3390/plants8110458 31671752 PMC6918393

[B47] Srivastava A. K. Lokhande V. H. Patade V. Y. Suprasanna P. Sjahril R. D’Souza S. F. (2010). Comparative evaluation of hydro-, chemo-, and hormonal-priming methods for imparting salt and PEG stress tolerance in Indian mustard (*Brassica juncea* L.). Acta Physiol. Plant 32, 1135–1144. doi: 10.1007/s11738-010-0505-y

[B48] Sung Y. Cantliffe D. J. Nagata R. T. Nascimento W. M. (2008). Structural changes in lettuce seed during germination at high temperature altered by genotype, seed maturation temperature, and seed priming. J. Am. Soc Hortic. Sci. 133, 300–311. doi: 10.21273/JASHS.133.2.300

[B49] Tavan M. Wee B. Fuentes S. Pang A. Brodie G. Viejo C. G.. (2024). Biofortification of kale microgreens with selenate-selenium using two delivery methods: Selenium-rich soilless medium and foliar application. Scientia Hortic. 323, 112522. doi: 10.1016/j.scienta.2023.112522

[B50] Teng Z. Luo Y. Pearlstein D. J. Wheeler R. M. Johnson C. M. Wang Q.. (2023). Microgreens for home, commercial, and space farming: a comprehensive update of the most recent developments. Annu. Rev. Food Sci. Technol. 14, 539–562. doi: 10.1146/annurev-food-060721-024636 36525689

[B51] Trod B. S. Buttarelli M. S. Stoffel M. M. Céccoli G. Olivella L. Barengo P. B.. (2023). Postharvest commercial quality improvement of broccoli (*Brassica oleracea* L.) after foliar biofortification with selenium. Crop Sci. 63, 784–800. doi: 10.1002/csc2.20904

[B52] Viltres-Portales M. Sánchez-Martín M. J. Llugany M. Boada R. Valiente M. (2024). Selenium biofortification of microgreens: Influence on phytochemicals, pigments and nutrients. Plant Physiol. Biochem. 206, 108283. doi: 10.1016/j.plaphy.2023.108283 38142664

[B53] Voutsinos O. Mastoraki M. Ntatsi G. Liakopoulos G. Savvas D. (2021). Comparative assessment of hydroponic lettuce production either under artificial lighting, or in a mediterranean greenhouse during wintertime. Agric. 11, 503. doi: 10.3390/agriculture11060503

[B54] Yemm E. W. Willis A. (1954). The estimation of carbohydrates in plant extracts by anthrone. Biochem. J. 57, 508. doi: 10.1042/bj0570508 13181867 PMC1269789

